# Black Spherical Silica with Controlled Carbon Content for High-Frequency PPE-Based Copper-Clad Laminates: Dielectric Properties and GHz Communication Applications

**DOI:** 10.3390/nano16080468

**Published:** 2026-04-15

**Authors:** Yingying Chen, Yingchun Guo, Shouquan Sun, Peisong Tang, Takeshi Yanagihara, Bin He

**Affiliations:** 1 Zhejiang Third Age Material Technology Co., Ltd., Huzhou 313000, China; cyy@zjtat.com (Y.C.);; 2 Department of Materials Engineering, Huzhou University, Huzhou 313000, China

**Keywords:** semiconductor packaging, black spherical silica, copper-clad laminate, dielectric properties

## Abstract

**Featured Application:**

The black spherical silica developed in this study exhibits well-controlled particle size distribution (D50 ≈ 2.0 μm), low moisture content (<0.1%), high electrical insulation (volume resistivity ∼10^13^ Ω·cm), and excellent dispersion stability. These properties make it suitable as a functional inorganic filler for high-frequency semiconductor packaging substrates, particularly for applications requiring both effective light-shielding capability (L* as low as 20.1) and low dielectric loss (D_f_ ≤ 0.0017 at 1–10 GHz). The findings provide practical guidance for the design of black fillers used in copper-clad laminates for 5G/6G communication devices.

**Abstract:**

This study utilized polysiloxane as the raw material to successfully prepare black spherical silica fillers with varying internal carbon content. Through different thermal treatment processes, a dense silica layer was formed on the particle surface, while the internal hydrocarbon groups were thermally decomposed into carbon. Four types of spherical silica with different carbon contents were systematically characterized in terms of particle size distribution (D50 ≈ 2.0 μm, D100 < 5 μm), scanning electron microscopy morphology, moisture content (<0.1%), specific surface area (~1.0–1.1 m^2^/g), true density (~1.90–1.97 g/cm^3^), carbon content, blackness (L* values), and volume resistivity. The results indicate that the prepared black spherical silica exhibits a narrow particle size distribution, low moisture content, and high electrical insulation properties. Furthermore, the prepared black spherical silica was used as a filler in a polyphenylene oxide-based binder system to fabricate copper-clad laminates (CCLs), and their dielectric properties were systematically investigated. The study found that at electric field frequencies of 1 GHz and 10 GHz, the dielectric constant (D_k_) and dielectric loss (D_f_) of CCLs prepared with fillers containing less than 5% carbon remained largely consistent with those of carbon-free control samples. However, the D_f_ of CCLs prepared with fillers containing 9.00% carbon increased nearly tenfold, indicating that when the internal carbon content of the filler exceeds a certain threshold, it adversely affects the high-frequency dielectric properties of the copper-clad laminate.

## 1. Introduction

Moore’s Law has guided the development of the global semiconductor industry for over five decades since it was proposed by Gordon Moore in 1965. It states that the number of transistors on an integrated circuit doubles approximately every 18 to 24 months, which has become a core guideline for the iterative upgrade of semiconductor technology. Over the past fifty years, the number of transistors integrated on semiconductor chips has largely followed this trajectory, driving the continuous miniaturization and performance improvement of electronic devices [1,2,3]. For many years, the semiconductor industry has relied primarily on innovations in process technology to integrate more transistors onto smaller chips, advancing from the 10 μm node in 1970 to TSMC’s mass-production 3 nm node in 2022. However, as transistor dimensions approach physical limits, the pace of Moore’s Law has slowed significantly, posing serious challenges to further enhancing chip performance through process scaling [4,5].

Against this backdrop, advanced packaging technology has emerged as a critical pathway to sustaining improvements in chip performance, enhancing integration density [6], and increasing energy efficiency. Concurrently, the explosive growth in computational power demand driven by applications such as artificial intelligence and autonomous driving has shifted competition in the semiconductor industry from a transistor density race to a packaging capability war. Consequently, mastery of high-end packaging technology now represents the strategic high ground for next-generation chip development. Compared to traditional packaging technologies such as wire bonding, advanced packaging technologies (including 2.5D/3D IC, fan-out wafer-level packaging, system-in-package, etc.) offer significant advantages such as miniaturization, thinner profiles, high integration density, low power consumption, and multifunctional integration. These advantages demonstrate substantial potential for improving chip performance, optimizing device form factors, and reducing overall manufacturing costs, making them widely applicable in high-end electronic products [7,8].

In semiconductor packaging materials, inorganic fillers play a crucial role in adjusting the comprehensive performance of packaging composites, typically accounting for 60~90% of the total composition of epoxy molding compounds and copper-clad laminate substrates [9,10,11,12,13,14,15]. Among various inorganic fillers, spherical silica is particularly favored in high-end packaging applications due to its excellent comprehensive properties, including high packing density, good flowability during processing, low coefficient of thermal expansion (CTE), minimal internal stress generation, and excellent dielectric properties [16,17]. Its primary function in packaging materials is to adjust the CTE mismatch among different materials (such as chip, substrate, and copper foil) within the packaging structure, thereby effectively preventing warpage, cracking, and interface detachment caused by thermal cycling during chip operation and improving the long-term reliability of electronic devices [18,19]. With the rapid advancement of 5G/6G communication and high-performance computing technologies, more comprehensive and stringent demands have been placed on packaging materials, especially for those requiring a specific black appearance. In addition to the basic requirements of low dielectric loss, high thermal conductivity, and good electromagnetic compatibility, these black packaging materials must also possess excellent light-blocking capability, weather resistance, chemical stability, and aesthetic appeal to meet the application needs of high-end electronic devices [20,21]. Traditionally, carbon black has been widely used as a blackening agent to achieve functions such as light shielding, laser marking, and anti-aging in packaging materials [22]. However, carbon black is inherently conductive, and its addition will significantly compromise the electrical insulation performance of packaging materials, making it difficult to meet the requirements of high insulation and low dielectric loss for advanced packaging substrates [23,24,25,26,27,28]. Therefore, developing a black filler that combines effective light-blocking properties with the superior electrical insulation and dielectric characteristics of white spherical silica has become an important research direction in the field of semiconductor packaging materials.

In this study, black spherical silica fillers with varying carbon contents were prepared using polysiloxane as the raw material through a controllable thermal treatment process. The preparation process involved thermally decomposing the internal organic components of polysiloxane into carbon under an inert atmosphere to ensure the retention of carbon in the silica particles, followed by calcination in an oxidizing gas environment to form a dense silica layer on the particle surface, which enhances the stability and electrical insulation performance of the fillers. By precisely controlling the calcination temperature and holding time during the heat treatment process, we successfully obtained three types of black spherical silica fillers with different carbon content gradients, as well as a carbon-free control group. The basic physical and chemical properties of these four types of spherical silica, including particle size distribution, morphology, moisture content, specific surface area, true density, carbon content, blackness, and volume resistivity, were systematically characterized. Furthermore, composite materials were fabricated using polyphenylene ether resin as the matrix and the prepared spherical silica as fillers, and the dielectric constant (D_k_) and dielectric loss (D_f_) properties of the resulting copper-clad laminates were investigated in detail to clarify the influence of filler carbon content on the dielectric performance of packaging substrates.

## 2. Materials and Methods

### 2.1. Preparation and Characterization of Black Spherical Silica

Materials and preparation steps were conducted as follows.

Materials

The following materials were used in this study. Polyphenylene ether (PPE) resin (SA9000, SABIC, Houston, TX, USA) served as the primary matrix resin. Epoxy resin (EBA-65HD, Shanghai Huayi, Shanghai, China) was used as a co-matrix to enhance processability. Black spherical silica fillers (DQM2405, CQM2405, and HQM2405 were synthesized in-house and commercially available through Zhejiang Third Age Material Technology Co., Ltd., Huzhou, China) were used as functional fillers. Toluene (analytical grade, Macklin, Shanghai, China) was employed as a solvent. m-Phenylenediamine (analytical grade, Aladdin, Shanghai, China) served as a curing agent for the epoxy resin. Styrene (analytical grade, Aladdin, China) was added as a reactive diluent. Trioctyl phosphate (analytical grade, Macklin, China) acted as a plasticizer to improve flexibility. Methyl ethyl ketone peroxide (analytical grade, Macklin, China) was used as an initiator for polymerization. For filler surface modification, KBM-503 (3-methacryloxypropyltrimethoxysilane, Shin-Etsu Chemical Co., Ltd., Tokyo, Japan) was used as a silane coupling agent. For glass fabric treatment, OFS-6032 (3-glycidoxypropyltrimethoxysilane, Macklin, China) was used as a silane coupling agent. Electronic-grade 2116 glass fabric (Taishan Fiberglass Inc., Tai’an, China) was used as the reinforcing substrate. HVLP (very low profile) copper foil (JX Nippon Mining & Metals, Tokyo, Japan) was used for lamination.

Preparation of BQM30

Add 80 parts by weight of methyltrimethoxysilane and 1 part by weight of 5% acetic acid to 1300 parts by weight of deionized water. Stir until the methyltrimethoxysilane dissolves to form a clear solution. Then, while stirring, add 100 parts by weight of 5% ammonia solution to the clear solution, completing the mixing within 1 min. Let it stand for at least 30 min. Then, filter and dry to obtain spherical polymethylsiloxane BQM30 powder.

Preparation of EQM2405

After grinding and dispersing the spherical polysiloxane BQM30 powder, place it in a tube furnace. First, under a nitrogen atmosphere with a nitrogen flow rate set to 300 mL min^−1^, raise the furnace temperature to 980 °C at a rate of 5 °C min^−1^. The temperature is held at 980 °C for 12 h to heat-treat the particles, causing the organic groups within them to thermally decompose into carbon. After cooling, the nitrogen atmosphere is switched to an air atmosphere, with the air flow rate set to mL min^−1^. The furnace temperature is then raised to 980 °C at a rate of 5 °C min^−1^, and the temperature is maintained for 9 h to perform heat treatment, thereby removing carbon from the particles and densifying their surfaces. The resulting sample is designated as EQM2405.

Preparation of DQM2405

After grinding and dispersing the spherical polysiloxane BQM30 powder, place it in a tube furnace. Set the nitrogen flow rate to 300 mL min^−1^. Under a nitrogen atmosphere, raise the furnace temperature at a rate of 5 °C min^−1^ to 980 °C. Hold the temperature for 12 h to heat-treat the particles, causing the organic groups within them to thermally decompose into carbon. The resulting sample is DQM2405.

Preparation of CQM2405

After grinding and dispersing the spherical polysiloxane BQM30 powder, place it in a tube furnace. Under a nitrogen atmosphere, set the nitrogen flow rate to 300 mL min^−1^, raise the furnace temperature to 1150 °C at a rate of 5 °C min^−1^, and hold at this temperature for 12 h to heat-treat the particles, causing the organic groups within them to thermally decompose into carbon. After the holding period, the nitrogen atmosphere is switched to an air atmosphere, and the furnace temperature is lowered at a rate of 5 °C min^−1^ to 980 °C. The temperature is held at 980 °C for 9 h to remove carbon from the particle surface and densify the surface. The resulting sample is designated as CQM2405.

Preparation of HQM2405

After grinding and dispersing the spherical polysiloxane BQM30 powder, place it in a tube furnace. Under a nitrogen atmosphere, set the nitrogen flow rate to 300 mL min^−1^ and raise the furnace temperature to 1150 °C at a rate of 5 °C min^−1^. Hold the temperature for 12 h to heat-treat the particles, causing the organic groups within them to thermally decompose into carbon. The resulting sample is HQM2405.

Characterization Equipment

A series of professional characterization equipment was used to systematically test the physical and chemical properties of the black spherical silica samples. The phase composition of the samples was analyzed using an X-ray diffractometer (TD-3500, Dandong Tongda Technology Co., Ltd., Dandong, China). Particle size distribution was measured by a laser particle size analyzer (Bettersize 3000 Plus, Dandong Bettersize Instruments Ltd., Dandong, China). Specific surface area was determined with a specific surface area analyzer (DX400, JWGB, Beijing, China). True density was tested by a true density tester (G-DenPyc 3900M, Guoyi Quantum, Hefei, China). Carbon content was quantified using a high-frequency infrared carbon-sulfur analyzer (CS-900, Cexi Instruments, Beijing, China). Moisture content was measured by a Karl Fischer moisture titrator (ST-LS1, Santihongke, Zibo, China). Morphology was observed, and elemental analysis was conducted via field emission scanning electron microscopy (Apreo 2C, Thermo Fisher Scientific, Waltham, MA, USA) equipped with an energy-dispersive X-ray spectroscopy (EDS) system. Blackness was evaluated using a desktop spectrophotometer (DS-36D, Hangzhou Caipu Technology Co., Ltd., Hangzhou, China). Elemental distribution was analyzed by an EDS system (Quantax Compact 30, Bruker, Berlin, Germany). Volume resistivity was measured using a high-resistance meter (4339B, Agilent, Santa Clara, CA, USA).

### 2.2. Preparation and Characterization of Black Silica/Resin Composites

Materials and Formulation

The resin varnish was formulated with the following components by weight percentage: 15.96 wt% polyphenylene ether (PPE) resin (SA9000, SABIC, Riyadh, Saudi Arabia), 39.89 wt% modified black spherical silica, 15.96 wt% toluene (solvent), 18.62 wt% epoxy resin (EBA-65HD, Shanghai Huayi), 5.32 wt% m-phenylenediamine (curing agent), 2.66 wt% styrene (reactive diluent), 1.06 wt% trioctyl phosphate (plasticizer), and 0.53 wt% methyl ethyl ketone peroxide (initiator). The total composition sums to 100 wt% of the varnish mixture.

Experimental Section

The composite preparation process consisted of several sequential steps. First, each of the four spherical silicas was surface-modified with 0.5 wt% KBM-503 (relative to filler weight) in ethanol solution, stirred at 60 °C for 2 h, and dried at 80 °C for 4 h, yielding the modified fillers designated EQM2405-M, DQM2405-M, CQM2405-M, and HQM2405-M. Meanwhile, electronic-grade 2116 glass fabric was calcined at 450 °C for 30 min in a muffle furnace to remove surface paraffin sizing. The calcined fabric was then immersed in a 1 wt% OFS-6032 silane coupling agent solution in ethanol/water (95:5 *v/v*) for 30 min, followed by curing at 120 °C for 2 h to promote adhesion with the resin system. The resin varnish was prepared by combining the components in the following weight percentages: 15.96 wt% PPE resin, 39.89 wt% modified black spherical silica, 15.96 wt% toluene, 18.62 wt% epoxy resin, 5.32 wt% m-phenylenediamine, 2.66 wt% styrene, 1.06 wt% trioctyl phosphate, and 0.53 wt% methyl ethyl ketone peroxide. The mixture was shear-dispersed at 2000 rpm for 30 min using a high-speed disperser, followed by maturation at 25 °C for 6 h. The pretreated glass fabric was then impregnated with the matured resin varnish using a vacuum heating coater and dried at 90 °C for 8 min to produce prepreg sheets. Finally, eight plies of prepreg were stacked with HVLP copper foil on both sides and laminated at 200 °C under 3.5 MPa for 180 min using a vacuum hot press, yielding four copper-clad laminate (CCL) samples designated A, B, C, and D corresponding to fillers EQM2405-M, DQM2405-M, CQM2405-M, and HQM2405-M, respectively. Dielectric properties of the resulting CCLs were measured in accordance with IPC-TM-650 2.5.5.13 using a microwave network analyzer (5227A, Keysight Technologies, Beijing, China).

Characterization Equipment

Composite preparation and characterization were performed using the following equipment. A high-speed mixer (YLT-GH2, Changzhou Kaibang Drying Equipment Co., Ltd., Changzhou, China) was used for filler modification, while a high-speed disperser (SDF-1100, Hangzhou Youbao Electronics Co., Ltd., Hangzhou, China) was employed for resin varnish preparation. Prepreg fabrication was carried out using a vacuum heating coater (JQ-0012, Shanghai KMT Co., Ltd., Shanghai, China). Dielectric properties of the copper-clad laminates were measured with a microwave network analyzer (5227A, Keysight Technologies (China) Co., Ltd., Beijing, China).

## 3. Results

### 3.1. Appearance, Carbon Content, and Blackness Characterization

**Figure 1:**
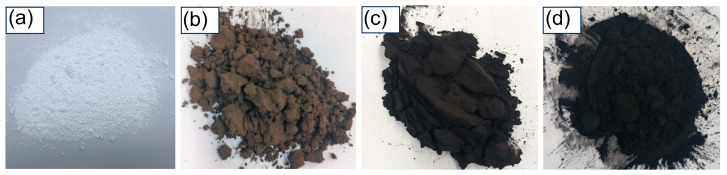
(**a**) EQM2405; (**b**) DQM2405; (**c**) CQM2405; (**d**) HQM2405 Appearance.

Figure 1 displays the macroscopic morphology of the four spherical silicas—EQM2405, DQM2405, CQM2405, and HQM2405. Visual inspection reveals a clear progressive darkening trend from left to right: EQM2405 is white, indicating that it has the lowest carbon content; DQM2405 appears brownish, which indicates a lower carbon content; CQM2405 is predominantly black with a faint brown tint, showing a moderate carbon content; and HQM2405 is deep black, suggesting the highest carbon content among the four samples. The carbon contents of the four samples were accurately determined with a high-frequency infrared carbon–sulfur analyzer, and the test results are listed in Table 1, showing a monotonic increase across the series: the carbon content of EQM2405 is 0.0089%, DQM2405 is 2.12%, CQM2405 is 4.95%, and HQM2405 reaches 9.00%, which is close to 10%. The test results confirm that the thermal treatment process can effectively regulate the carbon content of the spherical silica, achieving gradient control of carbon content.

To quantitatively evaluate the blackness of the four silica samples, each powder was uniformly dispersed in ethanol to form a suspension with a mass concentration of 5%, and the blackness index (L*) was measured with a desktop spectrophotometer based on the CIE 1976 color space standard. In the CIE 1976 color system, the L* value represents the lightness of the color, with a range of 0 (absolute black) to 100 (absolute white), so a lower L* value denotes a darker color [29]. The L* values of the four samples decrease from left to right: EQM2405 has an L* value of 93.3, DQM2405 is 28.7, CQM2405 is 23.6, and HQM2405 is 20.1, which is completely consistent with the visual observation trend. This result confirms that the carbon content is the key factor affecting the blackness of the spherical silica: higher carbon content can enhance the absorption of visible light by the silica particles, thereby making the powder color darker. The carbon particles formed by the thermal decomposition of polysiloxane are uniformly distributed inside the silica, which can effectively block the transmission of light, giving the silica a good black appearance and light-shielding performance.

### 3.2. XRD Phase Analysis

**Figure 2:**
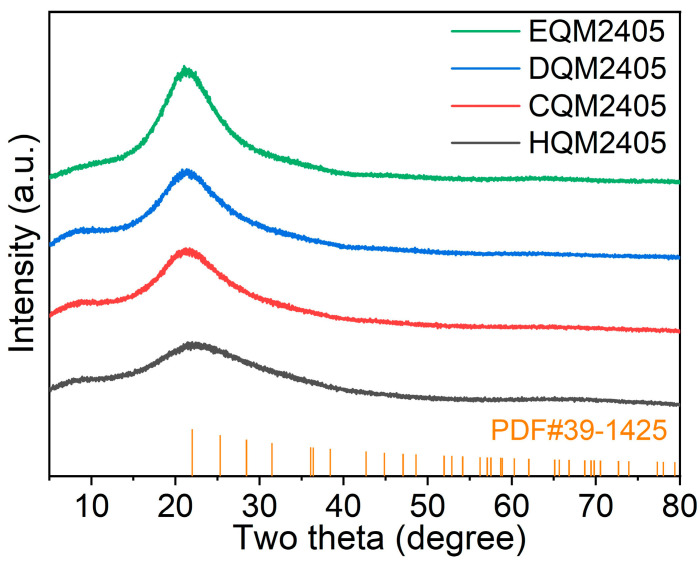
XRD Patterns of EQM2405, DQM2405, CQM2405, and HQM2405, together with the standard PDF card (No. 39-1425) of amorphous silica.

The crystal structures of the four spherical silica samples were systematically examined by X-ray diffraction (XRD), and the XRD patterns are presented in Figure 2. It can be clearly observed from the patterns that all four specimens exhibit a broad, diffuse halo centered at 2θ ≈ 22°, which is a typical characteristic peak of the amorphous Si–O–Si network structure of silica. This diffraction peak is consistent with the standard PDF card (No. 39-1425) of amorphous silica, indicating that the prepared spherical silica has an amorphous structure [30]. Amorphous silica has the advantages of low dielectric loss and good thermal stability, which makes it suitable for high-frequency semiconductor packaging applications.

It is worth noting that no obvious crystalline carbon phases (such as graphite, diamond, or amorphous carbon diffraction peaks) were detected in the XRD patterns of the four samples, even though the carbon content of HQM2405 reaches 9.00%. This phenomenon can be explained by two reasons: on the one hand, the carbon formed by the thermal decomposition of polysiloxane is uniformly dispersed inside the silica particles in the form of ultra-fine amorphous carbon nanoparticles, and the particle size of these carbon nanoparticles is smaller than the detection limit of XRD (usually less than 5 nm), so it is unavailable to be detected by XRD; on the other hand, the carbon content, even for HQM2405, is still relatively low, and the diffraction signal of carbon is weak, which is covered by the broad diffraction peak of amorphous silica. The XRD results confirm that the four spherical silica samples are entirely amorphous, and the introduction of carbon does not change the amorphous structure of silica, which is conducive to maintaining the excellent dielectric properties of silica.

### 3.3. Morphology and Particle Size Analysis

**Figure 3:**
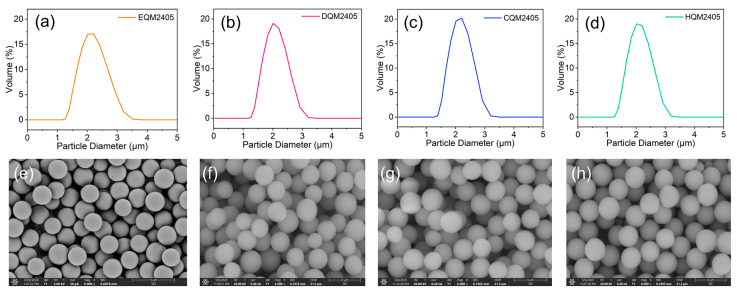
Laser particle size distribution of spherical silica. (**a**) EQM2405; (**b**) DQM2405; (**c**) CQM2405; (**d**) HQM2405. SEM image of spherical silica. (**e**) EQM2405; (**f**) DQM2405; (**g**) CQM2405; (**h**) HQM2405.

Figure 3a–f present the laser particle-size distributions and SEM micrographs of the four samples, which can intuitively reflect the particle size characteristics and morphological features of the samples. The laser particle size distribution curves show that the particle size distributions of the four samples are all narrow, and the size distributions closely match the morphological observations under SEM. The median diameter (D50) of each powder is approximately 2.1–2.2 µm. The maximum particle size (D100) of all samples is consistently below 5 µm. This indicates that the particle size of the silica is well controlled, with no noticeable large particles or agglomerates.

The SEM micrographs (magnification 6000×) reveal that all four samples have high sphericity, smooth surfaces, and uniform particle dimensions across the series. There are no obvious defects such as cracks, pores, or impurities on the particle surface, which indicates that the thermal treatment process does not damage the spherical morphology of the silica particles. The high sphericity of the silica particles is beneficial for improving the packing density of the filler in the resin matrix, reducing the internal voids of the composite material, and thus contributing to lower dielectric loss and a more stable dielectric constant in the resulting copper-clad laminate. Furthermore, comparative analysis shows that varying carbon content exerts no discernible influence on the particle morphology or size control of the black spherical silica, which is crucial for ensuring the consistency of the composite material performance when adjusting the carbon content to achieve different blackness requirements.

### 3.4. EDS Element Distribution Analysis

**Figure 4:**
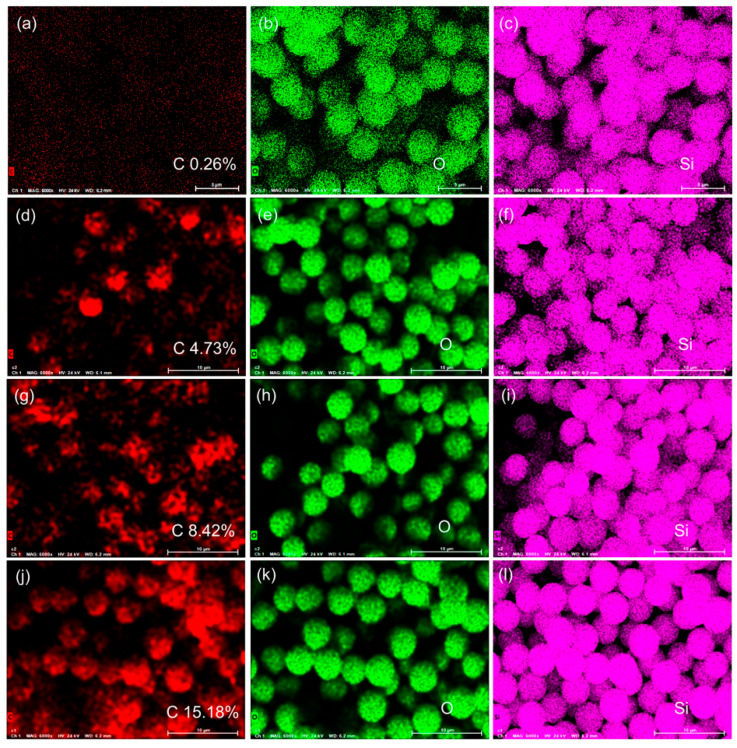
EDS mapping of spherical silica. (**a**–**c**) EQM2405; (**d**–**f**) DQM2405; (**g**–**i**) CQM2405; (**j**–**l**) HQM2405.

To clarify the spatial distribution and uniformity of carbon within the black spherical silica particles, EDS mapping tests were carried out on the four samples. The results are shown in Figure 4. EDS mapping is a powerful tool for analyzing the elemental distribution of materials with high spatial resolution, and the intensity of the characteristic signal of each element is positively correlated with the content of the element in the corresponding area. The EDS mapping results reveal distinct carbon distribution patterns across the four samples, which are consistent with the carbon content test results in Table 1.

For the carbon-free control sample EQM2405 (carbon content 0.0089 wt%), EDS mapping exhibits no discernible carbon signal above background noise, with carbon distribution appearing essentially featureless, consistent with its negligible carbon content. For DQM2405 (carbon content 2.12%), the EDS mapping shows an uneven carbon distribution with localized bright spots, indicating that the carbon elements are mainly concentrated in certain areas inside the silica particles, and the dispersion degree is relatively low. For CQM2405 (carbon content 4.95%), the EDS mapping displays increased bright spots and reduced dark areas compared with DQM2405, suggesting that the carbon content increases and the carbon elements are more uniformly dispersed inside the particles. For HQM2405 (carbon content 9.00%), the EDS mapping shows the most uniform carbon distribution with the highest signal intensity, which indicates that the carbon elements are uniformly distributed throughout the silica particles, forming a uniform carbon-silica composite structure. The gradual improvement in carbon dispersion with increasing carbon content can be attributed to the more complete thermal decomposition of the polysiloxane precursor during the synthesis process. Under heat treatment conditions involving higher temperatures, a greater proportion of the hydrocarbon groups in polysiloxanes undergo pyrolysis. This generates a greater quantity of amorphous carbon nanoparticles, which, due to the spatially confined environment within the silica matrix, become more uniformly distributed throughout the particles. This trend is consistent with the EDS mapping results (Figure 4), where the carbon signal intensity and uniformity progressively increase from EQM2405 to HQM2405.

### 3.5. True Density, Specific Surface Area, and Moisture Analysis

**Figure 5:**
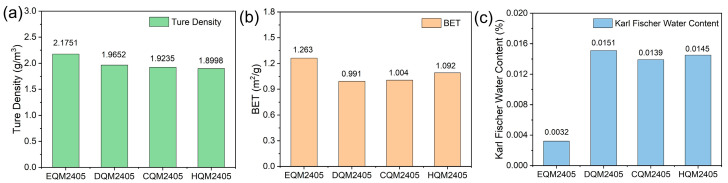
(**a**) Comparison of true density for four types of spherical silica; (**b**) Comparison of specific surface area; (**c**) Comparison of Karl Fischer moisture content.

The true density, specific surface area, and moisture content are important physical properties of inorganic fillers, which have a significant impact on the processing performance and comprehensive properties of composite materials. The true density affects the packing density of the filler in the resin matrix, the specific surface area is related to the interface bonding between the filler and the resin, and the moisture content can affect the dielectric properties and long-term stability of the composite material. The test results of the four spherical silica samples are shown in Figure 5, and the statistical analysis shows that the test results have good repeatability with a relative error of less than 5%.

The true density of the four samples was approximately 1.92 g/cm^3^ (±10%), with slight fluctuations: EQM2405 has a true density of 2.1751 g/cm^3^, DQM2405 is 1.9235 g/cm^3^, CQM2405 is 1.8998 g/cm^3^, and HQM2405 is 1.9652 g/cm^3^. The slight difference in true density is due to the difference in carbon content: carbon has a lower true density (about 1.2 g/cm^3^) than silica (about 2.2 g/cm^3^), so when the carbon content is moderate (CQM2405), the true density of the composite particles is slightly lower; when the carbon content is higher (HQM2405), the uniform dispersion of carbon may reduce the internal pores of the silica particles, leading to a slight increase in true density. However, overall, within the tested carbon content range of up to 9.00 wt%, the carbon content has no significant effect on the true density of the silica particles, and the true density of all samples is within a narrow range (1.90–1.97 g/cm^3^), which is conducive to the consistent processing performance of the composite material.

The specific surface area of the four samples was about 1.11 m^2^/g (±20%): EQM2405 has a specific surface area of 1.263 m^2^/g, DQM2405 is 0.991 m^2^/g, CQM2405 is 1.004 m^2^/g, and HQM2405 is 1.092 m^2^/g. The specific surface area of spherical silica is mainly determined by the particle size and surface roughness. Since the particle size of the four samples is basically the same and the surface is smooth (observed by SEM), the specific surface area is relatively close. The low specific surface area indicates that the silica particles have good flowability, which is conducive to improving the packing density in the resin matrix and reducing the viscosity of the resin varnish, facilitating the preparation of prepreg and copper-clad laminate.

### 3.6. Volume Resistivity Analysis

Volume resistivity is a fundamental measure of a material’s ability to resist the flow of electric current through its bulk, and serves as a key indicator for evaluating the electrical insulation performance of fillers used in semiconductor packaging applications. It directly determines the scope of application for that filler in semiconductor packaging. The volume resistivity of the four types of spherical silica samples was measured using a high-resistance meter, and the white spherical silica with the same particle size (EQM2405) was used as a reference sample. The test results are shown in Table 2. It can be seen from Table 2 that the volume resistivity of the four spherical silica samples is in the same order of magnitude (approximately 10^13^ Ω·cm) as that of the white spherical silica reference sample. The volume resistivity of EQM2405 is 7.0617 × 10^13^ Ω·cm, while the volume resistivity of CQM2405 is 7.1426 × 10^13^ Ω·cm, DQM2405 is 7.3731 × 10^13^ Ω·cm, and HQM2405 is 7.5278 × 10^13^ Ω·cm. The slight increase in volume resistivity with the increase of carbon content may be due to the more uniform dispersion of carbon in the silica matrix, which reduces the internal defects of the particles and improves the electrical insulation performance. This result indicates that although carbon elements are present in the black spherical silica, their form (ultra-fine amorphous carbon nanoparticles uniformly dispersed in the silica matrix) does not significantly affect the material’s insulating properties, which solves the problem that traditional carbon black fillers reduce the electrical insulation performance of packaging materials.

The excellent electrical insulation performance of the black spherical silica is mainly due to the dense silica layer on the particle surface and the uniform dispersion of carbon. The silica layer acts as an insulating barrier, preventing the formation of conductive paths between carbon particles, while the ultra-fine carbon nanoparticles are isolated from each other by the silica matrix, so they cannot form a continuous conductive network. This structure ensures that the black spherical silica has both good blackness and excellent electrical insulation performance, meeting the requirements of high-end semiconductor packaging materials.

### 3.7. Dielectric Properties Analysis

The dielectric properties of copper-clad laminates are crucial for their application in high-frequency semiconductor packaging, as they directly affect signal transmission speed and signal integrity of electronic devices. In particular, the relative permittivity (D_k_), also known as the dielectric constant, quantifies the material’s ability to store electrical energy under an alternating electric field, reflecting the extent of molecular polarization. A higher D_k_ generally leads to a slower signal-propagation velocity. The dissipation factor (D_f_), or loss tangent (tan δ), represents the inherent energy loss as electromagnetic waves propagate through the material. This loss arises from dipole relaxation and conductance loss, both of which convert electromagnetic energy into heat. With the development of 5G/6G communication technology, the working frequency of electronic devices is increasing, and higher requirements are put forward for the low dielectric constant and low dielectric loss of packaging substrates. The dielectric properties of the four copper-clad laminates A, B, C, and D (corresponding to EQM2405, DQM2405, CQM2405, and HQM2405 fillers, respectively) at 1 GHz and 10 GHz were tested, and the results are summarized in Table 3.

**Figure 6:**
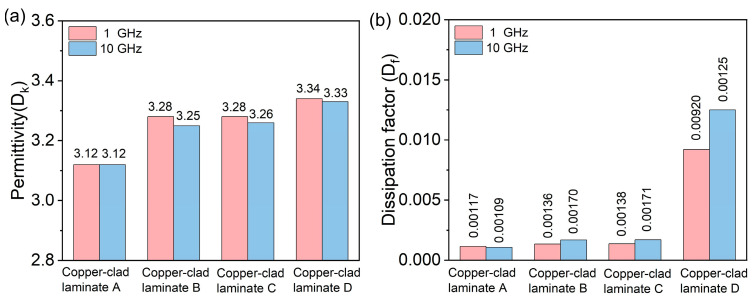
Dielectric properties of copper-clad laminates. (**a**) dielectric constant (D_k_) at 1 GHz and 10 GHz; (**b**) dissipation factor (D_f_) at 1 GHz and 10 GHz.

It can be seen from Figure 6 that the relative permittivity (D_k_) of the four CCL samples remains essentially constant at 3.1~3.3, regardless of the test frequency. At 1 GHz, the D_k_ values of A, B, C, and D are 3.12, 3.28, 3.28, and 3.34, respectively; at 10 GHz, the D_k_ values are 3.25, 3.26, and 3.33, respectively. The slight increase in D_k_ with the increase of carbon content is due to the fact that the dielectric constant of carbon is higher than that of silica [31], so the increase of carbon content will lead to a slight increase in the overall dielectric constant of the composite material. However, the change range of D_k_ is very small (less than 3%), which indicates that the carbon content has little effect on the dielectric constant of the CCL samples within the test range. This limited variation can be attributed to two main factors: first, the carbon nanoparticles are uniformly dispersed and isolated within the continuous silica matrix, preventing the formation of continuous high-permittivity pathways; second, at carbon contents below 10 wt%, the volume fraction of carbon is sufficiently low that its contribution to the overall Dk remains modest, as described by classical composite mixing rules.

In contrast, the dissipation factor (D_f_) of the CCL samples increases markedly with the carbon content of the filler, and the increasing trend is more obvious at high frequencies. At 1 GHz, the D_f_ of laminate A (EQM2405, carbon content 0.0089%) is 0.00117, laminate B (DQM2405, carbon content 2.12%) is 0.00136, laminate C (CQM2405, carbon content 4.95%) is 0.00138, and laminate D (HQM2405, carbon content 9.00%) is 0.0092, which is about 7.8 times that of laminate A. At 10 GHz, the D_f_ of laminate A is 0.00109, laminate B is 0.00170, laminate C is 0.00171, and laminate D is 0.0125, which is almost 11.5-fold higher than that of laminate A. This indicates that when the carbon content is below 5% (DQM2405 and CQM2405), the D_f_ of the CCL samples remain stable at a low level (about 0.0010~0.0017), which meets the requirements of high-frequency packaging substrates; when the carbon content approaches 10%, the D_f_ increases sharply, which will seriously affect the signal transmission performance of the CCL samples at high frequencies.

Since the filler carbon content was the only variable in the experiment (the resin matrix, glass fabric, and preparation process were all the same), the rise in D_f_ can be attributed primarily to the increased carbon loading. The mechanism of D_f_ increase is as follows: although the carbon in the black spherical silica is amorphous, partial sp^2^ hybridization may occur during the thermal decomposition process, forming micro-conjugated domains. These micro-conjugated domains have certain conductivity, and under an alternating electric field, they will generate conductance loss and polarization loss, thereby elevating the dielectric loss of the composite material. When the carbon content is low (below 5%), the micro-conjugated domains are isolated from each other by the silica matrix, and the loss generated is very small; when the carbon content approaches 10%, the number of micro-conjugated domains increases significantly, and they are close to each other to form a “percolation network”, which greatly increases the conductance loss and polarization loss, leading to a sharp increase in D_f_. In addition, the increase in D_f_ at high frequencies is due to the fact that the polarization response of the micro-conjugated domains cannot keep up with the change in the alternating electric field at high frequencies, resulting in increased polarization loss [32].

## 4. Discussion

The research results of this study have important implications for the development and application of functional inorganic fillers for semiconductor packaging, especially for the preparation of black insulating silica fillers with both light-shielding performance and high-frequency dielectric properties. With the miniaturization and high integration of electronic devices, the demand for black packaging materials with light-shielding performance is increasing (to avoid light interference of photoelectric devices). Meanwhile, high-frequency communication technologies (5G/6G) impose strict requirements on the low dielectric loss of packaging materials. The black spherical silica prepared in this study solves the contradiction between blackness/light-shielding performance and insulation/dielectric properties by means of in situ carbon doping and gradient content control, which is a breakthrough in the field of semiconductor packaging fillers and can be extended to the preparation of other black inorganic non-metallic fillers (e.g., alumina and boron nitride).

In addition, the preparation method of thermal decomposition of polysiloxane to realize in situ carbon doping and gradient control of carbon content has the advantages of a simple process, good repeatability, and easy industrialization, which is suitable for large-scale production and has important industrial application value. Compared with traditional physical blending of carbon black and silica, this method avoids the agglomeration of carbon black, poor dispersion, and reduced electrical insulation performance, and has obvious technical advantages.

Based on the research results of this study, the following future research directions are proposed:

The findings of this study open several avenues for future research. First, further optimization of carbon content and structure is warranted, particularly within the 5–9 wt% range, to identify the optimal balance between blackness and dielectric loss. The sp2/sp3 hybridization ratio of amorphous carbon could be precisely tuned by adjusting thermal treatment parameters (temperature, time, atmosphere), potentially reducing micro-conjugated domain formation and enabling black spherical silica with both high blackness and ultra-low dielectric loss. Second, advanced surface modification strategies beyond conventional silane coupling agents could be explored to further enhance filler-matrix interfacial bonding, potentially yielding additional reductions in dielectric loss and improvements in mechanical properties, such as flexural and impact strength. Third, mechanistic studies employing advanced characterization techniques—including TEM, XPS, and broadband dielectric spectroscopy—are needed to elucidate the size, distribution, and hybridization state of carbon nanoparticles within the silica matrix. Such investigations would support the development of quantitative structure–property relationship models linking carbon architecture to dielectric loss. Fourth, the application scope of the prepared black spherical silica could be extended to photoelectric device packaging, automotive electronics, and high-frequency printed circuit boards (PCBs), as well as light-shielding coatings and functional plastics, leveraging its combination of blackness and electrical insulation. Finally, hybrid filler systems combining black spherical silica with other low-dielectric-loss fillers (e.g., boron nitride, hollow silica) may offer synergistic property enhancements, enabling multifunctional composites that meet the demanding requirements of next-generation semiconductor packaging, including light-shielding capability, low dielectric constant, low dielectric loss, and high thermal conductivity.

## 5. Conclusions

Four spherical silicas with systematically graded carbon contents were synthesized and incorporated into PPE-based copper-clad laminates. Comprehensive characterization revealed that morphology, particle size, density, specific surface area, and moisture content remained essentially invariant across the series. Therefore, the carbon content was the sole discriminating parameter. The incorporation of carbon via in situ decomposition offers several distinct advantages. First, the uniformly dispersed carbon nanoparticles impart excellent blackness without compromising electrical insulation; all black spherical silica samples exhibit volume resistivity values (~10^13^ Ω·cm) comparable to those of carbon-free white silica. Second, this approach eliminates the agglomeration and dispersion issues commonly associated with physical blending of carbon black, ensuring consistent processing performance and composite quality. Third, at moderate carbon contents (≤5 wt%), the D_f_ of the resulting copper-clad laminates remains comparable to that of carbon-free control samples (0.00136–0.00171 at 1 GHz and 10 GHz), while simultaneously achieving effective light-shielding capability. However, once the carbon level approached 10 wt%, D_f_ rose sharply—an effect attributed to the emergence of micro-conductive pathways within the composite. Notably, the D_k_ values exhibited only a slight increase (from 3.24 to 3.34) across the same carbon content range, indicating that the dielectric constant is less sensitive to carbon loading compared to dielectric loss. These findings provide clear design guidance for advanced semiconductor packaging materials: maintaining carbon content below 5 wt% simultaneously affords effective light shielding and low dielectric loss, offering immediate engineering value for next-generation substrates.

## Figures and Tables

**Table 1:** Carbon Content and Blackness of EQM2405, DQM2405, CQM2405, and HQM2405.

Properties	EQM2405	DQM2405	CQM2405	HQM2405
Carbon Content (wt%)	0.0089	2.12	4.95	9.00
L* (Blackness)	93.3	28.7	23.6	20.1

**Table 2:** Volume Resistivity.

Number	Sample Name	Resistivity (Ω·cm)
1	EQM2405	7.0617 × 10^13^
2	CQM2405	7.1426 × 10^13^
3	DQM2405	7.3731 × 10^13^
4	HQM2405	7.5278 × 10^13^

**Table 3:** Dielectric Properties of A, B, C, and D.

Project	Copper-Clad Laminate A	Copper-Clad Laminate B	Copper-Clad Laminate C	Copper-Clad Laminate D
D_k_ (1 GHz)	3.12	3.28	3.28	3.34
D_f_ (1 GHz)	0.00117	0.00136	0.00138	0.00920
D_k_ (10 GHz)	3.12	3.25	3.26	3.33
D_f_ (10 GHz)	0.00109	0.00170	0.00171	0.0125

## Data Availability

The original contributions presented in this study are included in the article. Further inquiries can be directed to the corresponding author.

## Abbreviations

The following abbreviations are used in this manuscript:

SEM	Scanning Electron Microscope
CCLs	Copper-Clad Laminates
TSMC	Taiwan Semiconductor Manufacturing Company
CTE	Coefficient of Thermal Expansion
PPE	Polyphenylene Ether
CIE	Commission Internationale de l’Éclairage
XRD	X-ray Diffraction
EDS	Energy Dispersive Spectroscopy
